# Contributions of Fat and Fatty Acids Intake to the Latin American Diet: Results of ELANS Study

**DOI:** 10.3390/nu16223940

**Published:** 2024-11-19

**Authors:** Lilia Yadira Cortés Sanabria, Marianella Herrera-Cuenca, Martha Cecilia Yépez García, Pablo Hernández, Guillermo Ramírez, Maura Vásquez, Yaritza Sifontes, María Reyna Liria-Domínguez, Attilio Rigotti, Mauro Fisberg, Agatha Nogueira Previdelli, Irina Kovalskys, Maritza Landaeta-Jiménez, Georgina Gómez

**Affiliations:** 1 Departamento de Nutrición y Bioquímica, Pontificia Universidad Javeriana, Bogotá 110231, Colombia; 2 Centro de Estudios del Desarrollo, Universidad Central de Venezuela (CENDES-UCV), Caracas 1053, Venezuela; marianella.herrera@ucv.ve; 3 Fundación Bengoa, Caracas 1053, Venezuela; yaritza2sifontes@gmail.com (Y.S.); mlandaetajimenez@gmail.com (M.L.-J.); 4 Department of Nutrition and Health, Framingham State University, Framingham, MA 01702, USA; 5 Colegio de Ciencias de la Salud, Universidad San Francisco de Quito, Quito 170901, Ecuador; myepez@usfq.edu.ec; 6 Escuela de Nutrición y Dietética, Facultad de Medicina, Universidad Central de Venezuela, Caracas 1053, Venezuela; doctuscumliber@gmail.com; 7 Área de Postgrado en Estadística, Facultad de Ciencias Económicas y Sociales, Universidad Central de Venezuela, Caracas 1053, Venezuela; guillermo.ramirez.ucv@gmail.com (G.R.); mauralvasquez@gmail.com (M.V.); 8 Department of Nutrition and Health, Instituto de Investigación Nutricional, La Molina 15026, Peru; rliria@iin.sld.pe; 9 Centro de Nutrición Molecular y Enfermedades Crónicas, Departamento de Nutrición, Diabetes y Metabolismo, Escuela de Medicina, Pontificia Universidad Católica, Santiago 8330024, Chile; arigotti@med.puc.cl; 10 Centro de Excelencia em Nutrição e Dificuldades Alimentaes (CENDA), Instituto Pensi, Fundação José Luiz Egydio Setubal, Hospital Infantil Sabará, São Paulo 01228-200, Brazil; mauro.fisberg@gmail.com; 11 Departamento de Pediatria, Universidade Federal de São Paulo, São Paulo 04023-061, Brazil; 12 Facultad de Ciencias de la Salud, Universidad Autónoma de Chile, Av. Pedro de Valdivia 425, Providencia 7500912, Chile; agatha.usp@gmail.com; 13 Carrera de Nutrición, Facultad de Ciencias Médicas, Pontificia Universidad Católica Argentina, Buenos Aires C1107AAZ, Argentina; ikovalskys@gmail.com; 14 Departamento de Bioquímica, Escuela de Medicina, Universidad de Costa Rica, San José 11501-2060, Costa Rica; georgina.gomez@ucr.ac.cr

**Keywords:** dietary fats, dietary intake, saturated fatty acids, monounsaturated fatty acids, polyunsaturated fatty acids, trans fatty acids, diet, Latin America, ELANS

## Abstract

Background/Objectives: Fats, although essential for the proper functioning of the body, have been linked to an increased risk of developing chronic diseases, especially cardiovascular disease. The aim of this study was to evaluate the intake of total fat and its components (saturated, monounsaturated, polyunsaturated, and trans fats) in men and women aged 15 to 65 years of the urban population in eight Latin American countries. Methods: Survey data were collected from the Latin American Study of Nutrition and Health (ELANS, by its acronym in Spanish), an epidemiological study, including 9218 subjects from Argentina, Brazil, Chile, Colombia, Costa Rica, Ecuador, Peru, and Venezuela. Results: In general, 76.2% of the subjects consumed fat within normal ranges (20–35% of the total caloric value (TCV)). When analyzing its components, a majority of the subjects consumed saturated and polyunsaturated fats within the recommended ranges. However, 94.5% of the population does not comply with the recommended maximum intake of monounsaturated fatty acids (MUFA) (10–20%), and only 57.5% comply with the intake recommendation for trans fatty acids (TRANS) (0–2%). Likewise, on average, women had a significantly higher intake of all types of fat compared to men. Finally, the average fat intake by age indicates that regardless of the age range, the consumption of all fats except MUFA and TRANS are within the recommended ranges, with MUFA being slightly below and TRANS above. Conclusions: Even though the fat intake of the population falls within the recommended range, it is necessary to improve the quality by favoring the consumption of MUFA-rich foods regionally accepted, such as avocados, and reducing the consumption of TRANS.

## 1. Introduction

Dietary patterns encompass the balance, variety, and combination of foods and beverages that are routinely consumed. This includes all foods and beverages, whether prepared and consumed at home or outside the home [1]. This being so, the World Health Organization (WHO) recommends the adoption of a healthy lifestyle that reduces the risk of being seriously ill or of early death [2]. Within the healthy lifestyle, a healthy and balanced diet in macro and micronutrients is included.

Fats and lipids play a fundamental role in maintaining health. They are essential in the body’s energy reserve and are involved in important processes, such as the regulation of body temperature or the transport of nutrients, among others. Additionally, the quality and type of fat consumed can affect intellectual and mental capacity [1]. A high intake of saturated fats has been linked to cognitive decline, while the consumption of polyunsaturated fatty acids has beneficial effects in their prevention. As such, omega-6:3s are associated with better memory and a lower risk of cognitive decline [3] in diets with an adequate ratio (5:1) of polyunsaturated fatty acids (PUFA) [3]. On the other hand, lipids are essential to assimilating certain vitamins and for metabolism to function properly, so their importance is very prominent [4].

Among macronutrients, fat intake has long been a topic of interest and discussion in the scientific community. Early animal studies, dating back to the 1930s, reported a relationship between fat intake and the development of cardiovascular disease (CVD) [5]. A 1950s study by Keys et al. [6], reinforced this interest by correlating a high fat intake with increased plasma cholesterol, which, in turn, increases the risk of developing CVD. This hypothesis was later reinforced by a seven-country study, which pointed out that saturated fats were at the center of the public health problem caused by CVD [7]. Contradictory messages from the scientific community have distorted the general public’s understanding of the importance of quality fat as opposed to quantity. Despite widespread views derived from early studies that fat is the “bad guy”, researchers now agree that the intake of quality fats is instrumental in maintaining human health [8]. Despite extensive studies spanning several decades, controversy still exists about the effects of the different types of fatty acids on human health. Particularly those of saturated fatty acid (SFA) in chronic diseases, with a major emphasis on the risk of CVD [9].

Since the early 2000s, dietary recommendations have increasingly acknowledged the consequences of focusing on a low-fat diet. This has shifted mainstream views toward the importance of specific types of dietary fat [1]. It is often reported that SFAs raise low density lipoprotein (LDL) cholesterol levels, while monounsaturated fatty acids (MUFA) and PUFAs generally lower LDL cholesterol levels; being so, long-chain omega-3 fatty acids, such as eicosapentaenoic acid (EPA) (20:5n–3) and docosahexaenoic acid (DHA) (22:6n-3), are linked to lower triglyceride levels in hypertriglyceridemic patients and a reduced risk of developing coronary heart disease (CHD), while dietary trans-fatty acids are associated with higher LDL cholesterol levels [1,9]. However, recent studies have shown that high saturated fat intake tends to increase large LDL particles and/or decrease small LDL particles. Conversely, limited studies have found that diets rich in PUFAs decrease both large and small LDL particles, compared to diets high in saturated fats. However, higher-fat diets containing a mix of different fatty acids have shown no significant differences in LDL particle size [10].

On the other hand, trans-fatty acids (TRANS) and unsaturated fats derived from either industrial processes or natural sources have been linked to an increased risk of CVD. Each year, more than 278,000 deaths worldwide are attributed to the consumption of industrial TRANS. WHO guidelines advise that adults should limit their intake of TRANS to less than 1% of their total daily energy, which equates to less than 2.2 g per day for a diet of 2000 calories [11]. TRANS are commonly found in processed foods like margarine, vegetable shortening, and ghee; fried foods, and various baked goods like cookies and pies; as well as in many street and restaurant foods. Naturally occurring TRANS are present in red meat and dairy products [11].

Considering the important role of fats in human health and disease processes, population dietary assessments should include a comprehensive analysis of fat intake, detailing both the total amount and the specific breakdown of each fatty acid. An excessive fat intake is linked to obesity and an increased risk of chronic diseases, while insufficient fat intake can lead to undernutrition, skin disorders, and immune system impairments [8].

Given the significant social impact of dietary advice, it is crucial that recommendations provided to the public are grounded in solid scientific evidence and take into account the reasons for which people, in general, choose to eat what they eat. From several decades ago until current research, taste, cost, social and food environments, and role modeling are key in the behaviors people express at the moment of choosing the foods they will ultimately eat [12]. Recent research has highlighted that the quality of fat and the replacement of certain fats are more critical for chronic disease prevention than the total fat intake (TFI), therefore, understanding what people eat will help in making better recommendations for improving the quality of consumed fats [13].

Finally, rising obesity rates in Latin American (LA) countries are closely linked to poor-quality diets for a significant portion of the population, highlighting the importance of analyzing fat intake. The ELANS data offer a unique opportunity to gather reliable information, as the foundation for programs and policies, aimed at improving public education and enhancing population well-being. Therefore, this study aims to describe the consumption of total fats and their subtypes, identify their dietary sources, and assess their adherence to the recommendations of international organizations across eight Latin American countries, while also exploring differences based on sociodemographic variables.

## 2. Materials and Methods

### 2.1. Study Design

The Latin American Nutrition and Health Survey (ELANS) is a household-based, multicenter, cross-sectional study of nutrition and health surveillance [14]. The aim of the whole ELANS study was to evaluate the nutritional intake, physical activity levels, and anthropometric data of its participants. The ELANS was carried out simultaneously in eight Latin American countries (Argentina, Brazil, Chile, Colombia, Costa Rica, Ecuador, Peru, and Venezuela), from September 2014 to July 2015. The design and methodology of the ELANS study can be found in Fisberg et al. [14].

### 2.2. Sample

The study used a sample of 9218 participants, aged 15 to 65 years, from urban populations across eight LA countries. A randomized, complex, multistage sampling process was employed, with data stratified by geographical region, sex, age, and socio-economic status (SES). The sample size was calculated with a 95% confidence level and a margin of error of 3.49%. Sampling weights were applied for each country to ensure representativeness. SES was assessed using country-specific questionnaires, developed in accordance with legislative requirements or established local standards. For a detailed overview of the study’s structure and methodology, refer to Fisberg et al. [14].

### 2.3. Dietary Assessment

Trained interviewers used a two-face-to-face, 24 h dietary recall (R24h) to collect dietary intake data, including detailed information on all food and beverages, including alcoholic beverages, recipes, and supplements consumed. Following the multiple pass method (MPM) [15], interviews were conducted on two non-consecutive days.

A photographic album illustrating common household utensils and portion sizes was employed to quantify reported dietary intakes, with each album tailored to the specific context of the respective country. Data collected was converted into grams and milliliters by nutritionists trained in standardized data transformation methodologies. Subsequently, these measurements were translated into energy units. A food-matching standardization procedure was conducted by trained dietitians in each country using the Nutrition Data System for Research (NDS-R) software (version 2013), developed by the Nutrition Coordinating Center at the University of Minnesota [16].

Energy intake included quantification of macro and micronutrient intake, where usual fat intake and energy from food were determined using the multi-source method (MSM) (http://msm.dife.de/, accessed on 20 July 2024), an online tool developed by the European Prospective Investigation into Cancer and Nutrition (EPIC). The MSM is employed to estimate participants’ typical intake of nutrients, foods, and energy [17]. This technique, employed to convert individual intake data gathered from R24 h sessions into distributions reflecting usual intake patterns, facilitates a comprehensive assessment of dietary habits, enabling a more accurate understanding of nutritional intake among study participants.

The fat consumption record for this study included information on the type of fatty acids and cholesterol and the sources of fat intake. The types of fatty acids included were saturated, monounsaturated, polyunsaturated, and trans fatty acids.

Fat intake was assessed and evaluated against the US Institute of Medicine (IOM) Dietary Reference Intakes (DRI) standards [18], which establishes an estimate of total fat of 20–35% of total intake of calories, of which approximately 10% should be coming from SFA and 6–11% should be coming from PUFA, while the remaining percentage should come from MUFA. These estimates translate into ranges of frequent intake: 15 to 21 g per day for women and 21 to 34 g per day for men of SFA; 18 to 24 g per day for women and 25 to 39 g per day for men of MUFA; and 9 to 11 g per day for women and 12 to 17 g per day for men of PUFA; and <300 mg of cholesterol per day. There are no recommended values for TRANS, as this should be near zero [18,19].

### 2.4. Statistical Analysis

A descriptive statistics analysis was conducted for continuous variables as means, standard error of the mean (SEM), and confidence intervals. Categorical measures are presented as counts and percentages. Analyses of variance tests were performed to assess whether there are significant differences in the behavior of continuous variables in subpopulations defined by qualitative variable categories. With the purpose of describing the TFI, in terms of four of its most important components, SFA, MUFA, PUFA and TRANS fats, a decision tree was built based on the Chi-squared Automatic Interaction Detection (CHAID) segmentation. In all cases, tests were performed with a statistical significance level of 0.05. Microsoft Excel^®^ 2016 software and the IBM SPSS^®^ version 25 statistical packages were used for data loading and analysis. To evaluate the effect of sociodemographic factors (country, SES, gender, and age groups) on fat intake, a multivariate analysis of variance (MANOVA) model was used, supported by the Wilks Lambda statistic. The percentage contribution of several food groups to total fat intake and each of its components was also obtained.

### 2.5. Ethics

The ELANS protocol, which was registered at Clinical Trials (#NCT02226627) and approved by the Western Institutional Review Board (#20140605), also received approval from the ethical review boards of the participating institutions. Participants provided informed consent for their inclusion in the country-level study, and their confidentiality was maintained using identification codes instead of names. Data transfers were securely conducted through a file-sharing system.

## 3. Results

The sample characteristics are shown in Table 1. Overall, 9218 adults aged 15–65 completed the questionnaire. The sociodemographic characteristics of the participants, including age and socioeconomic status across countries were included to assess the variables presented in Table 1.

The overall analysis of fat intake in the ELANS countries showed that 76.2% of the subjects consumed fat within normal ranges (20–35% of the total caloric value (TCV)). 18.9% consumed more fat than the recommended amounts and 4.9% were below the recommended intake.

When broken down into categories (SFA, MUFA, PUFA, and TFA), a majority of the subjects consumed saturated and polyunsaturated fats within the recommended ranges. However, it stands out that 94.5% of the population does not comply with the recommended maximum intake of MUFA (10–20%) and only 57.5% comply with the intake recommendation for TFA (0–2%) (Table 1).

Regarding cholesterol intake, it was observed that 61.3% of the population met the consumption recommendation of <300 mg (Table 1).

A detailed analysis of the intake for each fatty acid revealed that regardless of country, age, sex, or socioeconomic status, the most consumed saturated fatty acids are the long-chain, palmitic (16:0), and stearic (18:0), followed by medium-chain lauric acid (12:0), and short chain butyric acid (4:0).

### 3.1. Average Daily Fat Intake by Sex and Age

The analysis revealed that, on average, women had a significantly higher intake of all types of fat compared to men (Table 2). Notably, the 95% confidence intervals for the average intake of TFI, as well SFA and PUFA, fell within the recommended ranges for both men and women (20–35% for TFI, 6–11% for PUFA, and 10% for SFA). However, the average intake of MUFA was below the recommended range for both sexes (15–20%), while the intake of TRANS exceeded the recommended levels (<1%).

The ANOVA test indicates that no significant differences are observed in the average consumption of MUFA type fats (*p* = 0.998), SAT (*p* = 0.205), and TRANS (*p* = 0.592), by age groups. However, it can be seen that the energy from PUFA shows a significant decrease as age increases (*p* < 0.001), finding that the Tukey test of multiple comparisons indicates a marked decrease between the age groups; the decrease is more advanced for ages 20–34 years vs. 35–49 years (*p* = 0.02) and 35–49 years vs. 50–65 years (*p* = 0.04). Additionally, for all age groups, the 95% confidence intervals for the average percentage of energy provided by TFI, SFA, and PUFA indicate that their behavior is within the range of recommendations, while MUFA is below the recommendations, and that of TRANS fats is above the range of recommendations (Table 3).

### 3.2. Average Daily Fat Intake by Socioeconomic Level

Regarding fat consumption by socioeconomic status (SES), the ANOVA test indicates that the average of TFI and all of its components is significantly differentiated by social classes (*p* < 0.05), with the exception of PUFA (*p* = 0.270), which does not differ between social classes. Tukey’s ad hoc tests show that the differences in the TFI, MUFA, SAT, and TRANS intakes between social classes are essentially determined by the fact that the middle class has a significantly higher intake when compared to the other classes: TFImedium vs. TFIhigh (*p* < 0.001), TFImedium vs. TFIlow (*p* < 0.001); MUFAmedium vs. MUFAhigh (*p* < 0.001), MUFAmedium vs. MUFAlow (*p* < 0.001); SATmedium vs. SAThigh (*p* < 0.001), SATmedium vs. SATlow (*p* < 0.001); TRANSmedium vs. TRANShigh (*p* = 0.021), and TRANSmedium vs. TRANSlow (*p* < 0.001).

Additionally for all social classes, the 95% confidence intervals for the average intake of TFI, SAT, and PUFA indicate that their behavior is within the range of recommendations while the consumption of MUFA fats is below the recommendations. TRANS fats is above the range of the recommendations (Table 4).

### 3.3. Average Intake by Country

**Figure 1:**
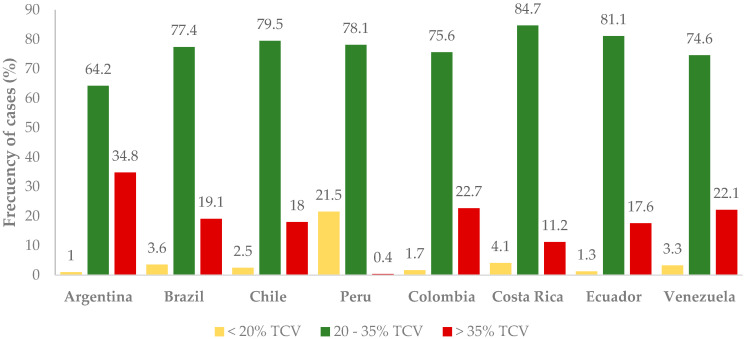
Percentage of energy from fat by country.

As shown in Figure 1, the majority of individuals (64.2% to 84.7%) meet the recommended intake levels, with Costa Rica standing out as the country where the population most adheres to the guidelines followed by Ecuador and Argentina as the countries with the lowest compliance. Notably, Argentina also has the highest percentage of individuals exceeding the recommended intake, followed by Colombia, Venezuela, and Brazil. In contrast, Peru has the highest percentage of individuals who tend to follow a low-fat diet (<20% TCV) (Figure 1).

Regarding PUFA intake, there is a notable pattern of high omega-6 fatty acid consumption and low omega-3 intake, resulting in an overall ω6:ω3 ratio of 10.2:1. Argentina has the highest ratio at 18.2:1, followed by Ecuador at 12.6:1 and Chile at 11.3:1. Conversely, Venezuela presents the most favorable ratio, with 7.1:1 (See Supplementary Materials).

**Figure 2:**
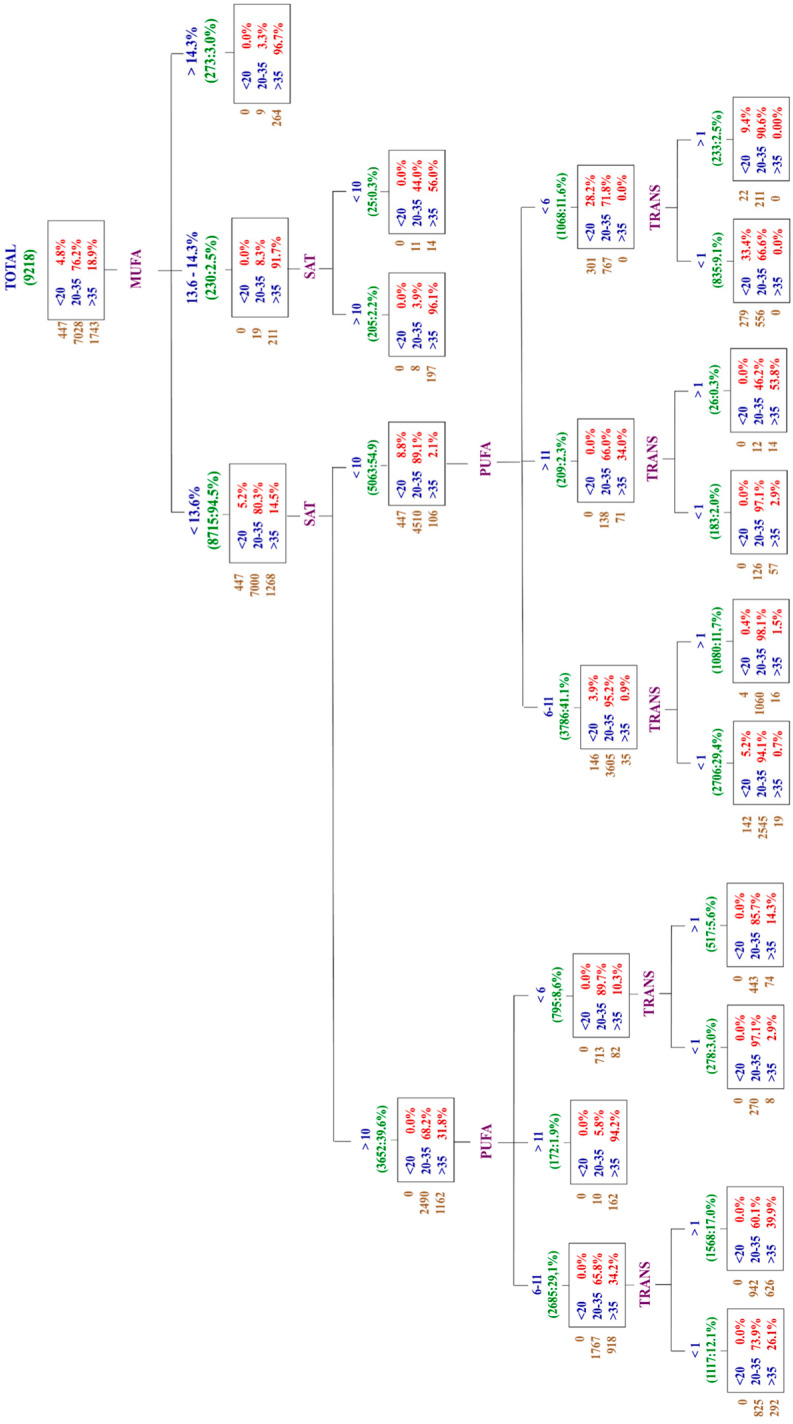
CHAID segmentation algorithm for fat intake adequacy in three level.

To describe the consumption of total fats, focusing on four key components—SFA, MUFA, PUFA, and TRANS fats—a decision tree was constructed using the CHAID segmentation algorithm (Figure 2). This resulted in 4 levels of segmentation and 14 terminal groups, as shown below.

The analysis reveals that 29.4% of the population consumes SFA, PUFA, and TRANS fats at levels within the recommended range, but their MUFA intake falls below the recommended levels. This group is followed by individuals who have excessive SFA and TRANS fat intake, with PUFA consumption within the recommended range, but with deficient MUFA intake (17%).

It should be highlighted that MUFA, in the decision tree, is identified as having the largest capacity to explain the total consumption of fats, because of the categories of consumption: low (<13.6%), within (13.6–14.3%) or higher than (≥14.3%) of the recommendations do establish the most important differences in the distribution of the participants according to their consumption of total fats (*p* < 0.001)

Then, within the first level of segmentation, there is a segment where almost all the participants in the study (91.3%) are characterized by a MUFA consumption below the recommendations. In this segment, the distribution shows a first small group with a low consumption of total fats (5.3%) a second large group with total fat consumption within the recommendations (82.4%) and a third group (12.3%) with a consumption of total fats above the recommendations. In the other two segments, with smaller number of individuals, when the MUFA consumption is within the recommendations, the consumption of total fats is high, whereas when MUFA intake is above the recommended values, total fats are also high.

In the second level of segmentation, when the MUFA intake is below the recommendations, it is shown that saturated fat intake introduces a new segmentation in two more groups. Interestingly, in the segment where MUFA is below recommendations, SAT fat intake derives into another two-group segmentation: the first, when MUFA is below the recommendations and the SAT are excessive, then the TFI is within the recommendations. The second is when MUFA is below the recommended values and SAT are within the recommendations, TFI fall within the recommendations, confirming the interesting findings of MUFA consumption, and the distribution of the different quality of fats.

To assess the impact of sociodemographic factors (country, SES, gender, and age group) on fat intake, a multivariate analysis of variance (MANOVA) was conducted, using Wilks’ Lambda statistic for support. Table 5 displays the significance of each sociodemographic factor, as measured by the Wilks’ Lambda statistic, in jointly explaining the behavior of the different types of fat under consideration. This allows for a comparison of the average intake patterns of SFA, MUFA, PUFA, and TRANS fats across the categories of each sociodemographic variable (gender, country, age group, and SES) separately.

These results indicate that sociodemographic variables significantly affect the intake of the components of TFI, SFA, MUFA, PUFA, and TRANS.

**Figure 3:**
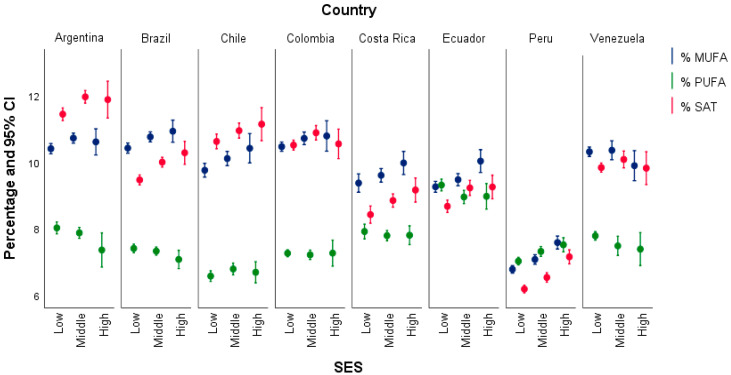
Percentage of energy from fat by country and socioeconomic status.

Analyzing the contribution of energy coming from the components of total fat by country and SEL, it can be observed that the averages of SFA and MUFA in all countries does not differ between upper and middle social classes, decreasing significantly in the lower social class. In Argentina, Brazil, and Venezuela, the energy coming from PUFA in the lower social class is relatively higher or similar than the middle class; in Colombia, Ecuador, and Chile there are not differences by social classes; Peru, in contrast to the other countries, presents a markedly different behavior: the energy contribution by the different components of fats is significantly different by social class, and it is also found in addition that the contribution determined by SFA is significantly lower than that corresponding as MUFA and PUFA, in all social classes. The energy provided by TRANS is similar in all countries except in Costa Rica and Brazil, which are the countries with the highest level of TRANS intake (Figure 3).

### 3.4. Food Sources of Fats

Finally, it is important to consider not only the consumption of fats and their components but also the sources of these fats. Therefore, a detailed analysis of food sources is presented below.

Across the whole sample, the primary source of TFI are vegetable oils, contributing between 29.5% in Ecuador and 15.2% in Chile. This is followed by unprocessed meats (beef, poultry, pork, and lamb), which contributed an average of 16.9%, with Brazil and Ecuador showing the highest percentages (20.4% and 20.8%, respectively) and Chile the lowest (11.9%). Cheese is the third major contributor to TFI, with significant variation across countries, ranging from 18.4% in Venezuela to 3.5% in Peru (Table 6).

As shown in Table 7, unprocessed meats are the primary source of saturated fats in all countries except Venezuela, where cheese is the leading source. Across the entire sample, cheese ranks as the second major source of saturated fats, with contributions ranging from 6.9% in Peru to 32.9% in Venezuela. Additionally, vegetable oils and processed meats are significant contributors, each accounting for approximately 10% of saturated fat intake on average.

In the entire sample, MUFA primarily come from unprocessed meat, contributing an average of 23.6%. The second main source is vegetable oils, with contributions ranging from 4.1% in Colombia to 21.0% in Peru (Table 8).

The mean contribution of vegetable oils to PUFA consumption is 38.6%, followed by a 7.8% of not-processed meat and 4.8% of salad dressings (Table 9).

The primary sources of TRANS are unprocessed meats, which account for 24.8% of trans fat intake for the overall sample. Industrial trans fats are mainly contributed by breads, which provide an average of 12.6%, with a range from 5.8% in Venezuela to 24.6% in Peru. For more details, see Table 10.

## 4. Discussion

To our knowledge, this is the first time that fat intake, its sources, and adherence to international recommendations have been evaluated at regional level. Our results indicate that while most of the population consumes fats within the recommended range, there are notable exceptions that warrant further attention.

### 4.1. Total and Saturated Fat

The data reveals that 76.2% of the overall ELANS population meets the recommendations for total fat intake, while 18.9% exceed the recommended levels, and a small percentage falls below the recommendations. Also, the segmentation, according to the quality of consumed fat, shows an interesting pattern, reporting that when MUFA are low, total fats might be above the recommended values. This variability highlights the need for targeted nutritional interventions to address both overconsumption and underconsumption of fats. Additionally, 55.5% of the population consumes more SFA fatty acids than recommended. These findings align with those from previous studies, such as Harika et al. [20], which reported that out of 40 countries analyzed, 25 (62.5%) met the recommended range of 20–35% of total caloric intake from fats, while 14 out of the remaining 15 countries had a mean total fat intake exceeding 35%. Furthermore, 67.5% of the countries had SFA intakes higher than the recommended level of >10% of total caloric intake [20].

Similarly, Eilander et al. [21] reported that 62.5% of the 24 European countries studied met the recommendations for total fat intake, but only 8.3% adhered to the SFA recommendations. The recommendation to limit dietary SFA intake to less than 10% of TCV has been a central focus of dietary guidelines. However, recent research has called this recommendation into question, suggesting that restricting SFA intake may not yield significant health benefits, particularly concerning cardiovascular diseases and total mortality [21,22].

The Cochrane Database systematic review by Hooper et al. [23], analyzed data from 11 randomized controlled trials involving 55,858 participants and found little to no effect of lowering saturated fat intake on mortality. In contrast, a systematic review and dose–response meta-analysis of prospective cohort studies demonstrated that each additional 10 g per day of SFA intake was associated with a 6% relative risk reduction for stroke [24]. Some studies have shown that replacing fats with carbohydrates is not necessarily associated with a lower risk of cardiovascular heart disease and may even be linked to increased total mortality, depending on the type of carbohydrates consumed. Conversely, replacing SFA with PUFAs has been shown to reduce the incidence of CVD [25].

A detailed analysis of saturated fatty acid intake [26] reveals that long-chain saturated fatty acids (palmitic and stearic) are the most commonly consumed, followed by medium-chain lauric and short-chain butyric acids. This is significant because not all saturated fats have the same effects on serum cholesterol or LDL cholesterol [26]. For example, butter and other dairy fats high in myristic acid tend to raise LDL cholesterol to a greater extent than beef fat, which contains palmitic and stearic acids and has a lesser impact on LDL cholesterol [27,28].

### 4.2. Mono and Polyunsaturated Fat

Only 2.5% of individuals consume the recommended amount of MUFA fatty acids. This is concerning, given the documented beneficial effects of MUFAs on cardiovascular health. For example, Schwingshackl et al. [29] found that individuals in the highest tertile of the MUFA:SFA ratio experienced an overall risk reduction of 11% for all-cause mortality, 12% for cardiovascular mortality, 17% for stroke, and 9% for cardiovascular events compared to those in the lowest tertile. However, it is important to highlight that a systematic review and meta-analysis published recently showed mixed results for MUFA and PUFA. Thirty studies [30] reported an inverse association between various fats, including MUFA and PUFA, and all-cause mortality, indicating potential health benefits. However, 22 studies found a positive association, primarily linked to SFA. The meta-analysis found no significant association between MUFA or PUFA intake and CVD or stroke morbidity, suggesting that while some studies indicate potential benefits, the overall impact of MUFA and PUFA on mortality and CVD events remains inconclusive [30].

The health effects of MUFAs can differ depending on their origin. Plant-derived MUFAs (MUFA-P) from sources such as olive oil, nuts, and salad dressings are associated with lower mortality. In contrast, animal-derived MUFAs (MUFA-A) from meat and dairy are linked to higher mortality. Replacing SFA, refined carbohydrates, or trans fats with MUFA-P has been shown to significantly reduce mortality. This is likely due to the bioactive nutrients and phytochemicals in plant sources, which improve metabolic health, lower blood pressure and obesity, and enhance antioxidant and anti-inflammatory effects, thereby reducing the risk of CVD, diabetes, and all-cause mortality [30].

Despite most findings linking olive oil with a reduced risk of morbidity and mortality, it is not the primary source of MUFAs in this study. Instead, unprocessed meat accounts for 23.6% of MUFA consumption in the overall sample; for example, in the case of oleic and palmitoleic acids present in chicken meat are related to endogenous synthesis or intestinal absorption of the diet [31]. One hypothesis is that the association of fatty acids with coronary heart disease (CHD) may vary depending on the food source. For instance, dairy intake is often associated with a protective or neutral effect on CHD risk, whereas meat intake, particularly red or processed meat, is generally linked to a higher risk of CHD [32].

In contrast with MUFA intake, almost 75% of participants met the recommendations for PUFA consumption. Both MUFAs and PUFAs have been negatively associated with CVD [33], kidney diseases [34], mental disorders [35], and cognitive impairment [36].

Epidemiological studies have demonstrated that adequate intake of linoleic acid (LA) can reduce plasma LDL-C levels. Dietary intervention studies have shown that replacing 5% of dietary SFA energy with ω-6 PUFAs can lower LDL-C by up to 10%, reduce the risk of coronary events by 13%, and decrease the risk of coronary death by 26% [37].

### 4.3. Trans Fat

It is noteworthy that 42.5% of the overall sample reported an intake of trans fats exceeding the recommended levels. This is concerning because numerous studies have established a direct association between the intake of industrial trans fats and the development of CVD, prompting national regulations to restrict their presence in food products [38]. This high intake may be related to the consumption of processed foods and partially hydrogenated oils commonly used in the food industry.

### 4.4. Food Sources

The main sources of total fat were vegetable oils and unprocessed meat, followed by cheese and processed meats. Vegetable oils, which are rich in essential fatty acids (EFAs), phytosterols, tocopherols, carotenoids, and phenolics, offer significant health benefits. They contribute to cardiovascular health by improving lipid profiles and reducing inflammation, have potential anti-cancer effects, and support chronic disease prevention through their diverse biological activities [36,39].

Red and processed meats have been associated with potential health effects due to their saturated fatty acids and cholesterol content [40]. It is crucial to promote the consumption of high-quality MUFA, such as those found in olive oil and avocados.

An analysis of fat intake by country and socioeconomic level reveals notable differences. For example, Costa Rica has the highest compliance with fat intake recommendations, whereas Argentina showed the lowest compliance. Previous analysis has shown that Argentina also reported the highest added sugar intake [41] and a lower overall diet quality index [42]. Furthermore, trans fat intake is higher in lower SES across most countries, which may be linked to increased consumption of processed and affordable foods.

As shown and discussed above, the consumption of fats in Latin America might not be what the audience expects: a diet high in fat, probably because people identify traditional foods as deep fried and loaded with oils, butter, whole dairy products, and more. However, the evidence shows an intake of total fats within the recommendations for the majority, but also a distribution of the type and quality of fats that is different from other populations. What is very interesting from the public health perspective is that taking into account the cultural traditions of the Latin American population, the new guidelines could explore giving more recommendations toward the use of the MUFA available in the region that people already use, such as avocados or fish. For instance, trying to incorporate olive oil might not be as successful as it is imported and expensive for households in the region.

It is relevant that emphasis should be placed on the qualitative aspects of the guidelines instead of the quantities for consumption. The new trends in guidelines are showing an important and better adherence when quality recommendations are made, instead of focusing on eliminating foods or diminishing the amounts of those.

This study has limitations, such as being a cross-sectional study; therefore, being able to establish causality is not possible. However, it has some strengths, such as having two 24 h recalls, the implemented standardized methodology across the eight countries, and the use of the multiple pass method. Consequentially, valuable information was obtained from this research, as it shows that the majority of the population has a fat consumption that falls between the limits of normal intake. It should be noted from the public policy perspective that some enhancement to the traditional sources of MUFA needs to be carried out in the region. While olive oil is not a traditional source of fats for the Latin American population, avocado, fish, and particularly sardines as well as some nuts are traditional sources, but they show low overall consumption. This is a relevant key issue for giving recommendations, building upon educational strategies and programs with the aim of enhancing a diverse, balanced, and healthier diet. It is true that people’s beliefs go toward “Latin American excessive consumption of fats” probably because of the use of deep-frying methods or other traditional use of fats; however, this study shows a need to better implement policies and programs that reinforce the consumption of quality traditional foods in our region, and we call on the regional authorities to reflect on this issue and go for recommendations that include the interesting cultural culinary roots the region has.

## 5. Conclusions

The findings of this study demystify the belief that Latin American diets are high in fats on the one hand and highlight the need to improve the quality of fats in the diet on the other. Interventions should aim to increase MUFA intake while reducing SFA and trans-fat consumption, acknowledging the traditional foods consumed in the region that would improve the MUFA consumption such as avocados. Additionally, it is crucial to consider the socioeconomic context when designing policies and educational programs to ensure that all sectors of society have access to healthy fat options. Further research is needed in the future to continue the evaluation of the fat intakes in the region as a key pivotal aspect for prevention of chronic diseases associated with nutrition.

## Figures and Tables

**Table 1:** Participants (n, %) meeting the recommended dietary intake for fats as percentage of total energy intake.

	Recommendation	n	%
% total fat	<20%	447	4.8
	20–35%	7028	76.2
	>35%	1743	18.9
% SFA	<10%	5112	55.5
	>10%	4106	44.5
% MUFA	<13.6%	8715	94.5
	13.6–14.3%	230	2.5
	>14.3%	273	3.0
% PUFA	<6%	1891	20.5
	6–11%	6893	74.8
	>11%	434	4.7
% TRANS	<1%	5303	57.5
	>1%	3915	42.5
Cholesterol	<300 mg	5652	61.3
	>300 mg	3566	38.7

**Table 2:** Percentage of energy from fat by sex.

Variable	Sex	Media	Standard Error	CI 95%	
				Lower Limit	Upper Limit
% Total fat	Male	29.37	0.087	29.20	29.54
	Female	30.30	0.082	30.14	30.46
	Total	29.85	0.060	29.74	29.97
% MUFA	Male	9.71	0.035	9.64	9.78
	Female	10.02	0.033	9.95	10.08
	Total	9.87	0.024	9.82	9.92
% PUFA	Male	7.42	0.028	7.37	7.48
	Female	7.70	0.028	7.65	7.76
	Total	7.57	0.020	7.53	7.61
% SFA	Male	9.47	0.039	9.39	9.54
	Female	9.92	0.037	9.85	9.99
	Total	9.70	0.027	9.65	9.76
% TRANS	Male	1.06	0.011	1.04	1.08
	Female	1.11	0.011	1.08	1.13
	Total	1.08	0.008	1.07	1.10

**Table 3:** Percentage of energy from fat by age group.

Variable	Sex	Media	Standard Error	CI 95%	
				Lower Limit	Upper Limit
% Total fat	15–19	30.24	0.160	29.93	30.56
	20–34	29.95	0.097	29.76	30.14
	35–49	29.73	0.114	29.51	29.96
	50–65	29.60	0.134	29.33	29.86
	Total	29.85	0.060	29.74	29.97
% MUFA	15–19	9.87	0.065	9.74	10.00
	20–34	9.87	0.039	9.79	9.95
	35–49	9.87	0.045	9.78	9.96
	50–65	9.88	0.055	9.77	9.99
	Total	9.87	0.024	9.82	9.92
% PUFA	15–19	7.77	0.054	7.66	7.87
	20–34	7.66	0.032	7.60	7.73
	35–49	7.52	0.037	7.45	7.59
	50–65	7.33	0.044	7.24	7.42
	Total	7.57	0.020	7.53	7.61
% SFA	15–19	9.77	0.072	9.63	9.92
	20–34	9.67	0.043	9.58	9.75
	35–49	9.66	0.052	9.56	9.76
	50–65	9.79	0.061	9.67	9.91
	Total	9.70	0.027	9.65	9.76
% TRANS	15–19	1.10	0.023	1.05	1.14
	20–34	1.07	0.012	1.05	1.10
	35–49	1.10	0.015	1.07	1.12
	50–65	1.08	0.018	1.05	1.12
	Total	1.08	0.008	1.07	1.10

**Table 4:** Percentage of energy intake from fat by socioeconomic level.

Variable	SES	Media	Standard Error	CI 95%	
				Lower Limit	Upper Limit
% Total fat	Low	29.40	0.182	29.04	29.75
	Middle	30.28	0.095	30.10	30.47
	High	29.62	0.085	29.46	29.79
	Total	29.85	0.060	29.98	29.97
% MUFA	Low	9.69	0.075	9.54	9.84
	Middle	10.03	0.038	9.96	10.11
	High	9.78	0.034	9.72	9.85
	Total	9.87	0.024	9.82	9.92
% PUFA	Low	7.52	0.062	7.40	7.64
	Middle	7.54	0.031	7.48	7.60
	High	7.60	0.028	7.54	7.65
	Total	7.57	0.020	7.53	7.61
% SFA	Low	9.40	0.083	9.24	9.56
	Middle	9.97	0.044	9.88	10.06
	High	9.56	0.037	9.49	9.64
	Total	9.70	0.027	9.65	9.76
% TRANS	Low	1.09	0.030	1.03	1.14
	Middle	1.16	0.014	1.13	1.19
	High	1.03	0.009	1.01	1.05
	Total	1.08	0.008	1.07	1.10

**Table 5:** Effect of each sociodemographic factor on daily fat intake.

Effect	Value	F	Dof Hypothesis	Dof Error	Sig
Sex	0.982	42.7	4	9159	0.000
SES	0.988	13.4	8	18,318	0.000
Age	0.991	6.8	12	24,233	0.001
Country	0.512	240.4	28	33,025	0.000
Sex × country	0.994	2.1	28	33,025	0.001
Age × country	0.987	1.4	84	36,180	0.007
SES × country	0.988	2.0	56	35,629	0.000

**Table 6:** Percentual contribution of food groups to total fats consumption (%).

Food Group	Argentina	Brazil	Chile	Colombia	Costa Rica	Ecuador	Peru	Venezuela	ELANS
Vegetable oils	18.4	19.6	15.2	17.4	23.8	29.5	28.5	21.2	21.7
Not-processed meat	15.4	20.4	11.9	18	16	20.8	15.3	17.6	16.9
Cheese	9.1	4.0	8.3	4.2	5.2	7.7	3.5	18.4	7.5
Processed meats	7.6	5.1	6.5	5.8	10.8	4.4	9.0	8.3	7.2
Refine grains (Breads and pasta)	12.6	4.4	9.4	2.5	2.7	3.6	2.9	3.4	5.2
Chips, Popcorn and other snacks	6.0	6.8	9.6	7.0	3.5	2.4	1.5	1.7	4.8
Eggs	4.5	2.6	4.2	5.8	4.2	2.9	5.7	3.2	4.1
Margarine	0.0	11.0	2.4	4.2	3.8	1.9	1.6	7.3	4.0
Butter	5.3	4.0	8.6	3.8	1.7	3.7	0.0	0.0	3.4
Milk, Whole and Whole Milk Products	2.1	5.5	1.7	6.7	2.0	3.0	3.5	2.6	3.4

**Table 7:** Percentual contribution of food groups to saturated fatty acids consumption (%).

Food Group	Argentina	Brazil	Chile	Colombia	Costa Rica	Ecuador	Peru	Venezuela	ELANS
Not-processed meats	16.6	24.7	11.1	20.4	18.1	26.4	24.9	19.3	20.2
Cheese	15.9	7.6	14.5	7.0	9.7	14.6	6.9	32.9	13.6
Vegetable oils	5.5	8.9	5.5	7.9	11.2	11.8	17.0	10.4	9.8
Processed Meat	10.1	9.8	12.5	12.4	13.2	8.2	4.0	6.5	9.6
Milk and milk products	3.4	9.2	4.1	11.4	5.5	7.9	9.6	5.4	7.0
Butter	9.5	7.4	15.2	7.0	3.0	0.0	2.3	0.0	5.5
Breads, Refined Grain	5.1	3.6	8.8	1.7	2.0	8.0	2.4	2.2	4.2
Eggs	4.0	2.4	3.6	5.2	4.3	3.1	6.3	3.1	4.0
Cakes, pies and cookies	5.1	1.5	2.4	1.0	2.9	2.5	2.8	1.2	2.4
Margarine	6.5	0.0	0.0	2.6	2.7	1.1	1.4	2.5	2.1

**Table 8:** Percentual contribution of food groups to monounsaturated fatty acids consumption (%).

Food Group	Argentina	Brazil	Chile	Colombia	Costa Rica	Ecuador	Peru	Venezuela	ELANS
Not-processed meat	24.8	29.8	15.5	27.2	20.4	26.1	27.8	17.3	23.6
Vegetable oils	12.2	16.6	11.2	4.1	16.7	21.0	20.9	14.0	14.6
Processed meat	6.3	6.9	8.1	8.1	10.7	6.2	2.1	4.3	6.6
Cheese, Processed	8.0	3.4	6.7	2.7	3.7	6.2	2.9	14.2	6.0
Eggs, Regular	5.5	3.1	4.9	6.6	4.9	3.7	7.3	3.7	4.9
Fats, margarine	0.0	7.2	2.8	5.1	5.4	3.1	1.4	13.5	4.8
Breads, White Bread, Regular	4.2	2.4	5.1	1.7	1.3	3.1	0.0	1.4	2.4
Butter, Regular	4.3	2.9	6.7	2.0	0.0	2.4	0.0	0.0	2.3
Avocado	0.0	0.0	9.2	1.3	1.9	1.1	2.9	0.0	2.1
Bread, white, sweet bread, regular	1.2	0.0	0.0	0.0	0.0	0.0	2.1	10.8	1.8

**Table 9:** Percentual contribution of food groups to PUFA consumption (%).

Food Group	Argentina	Brazil	Chile	Colombia	Costa Rica	Ecuador	Peru	Venezuela	ELANS
Vegetable oils	48.7	42.0	40.2	38.6	25.9	13.2	52.2	48.0	38.6
Not-processed meat	5.8	7.2	6.0	8.0	7.2	8.9	11.1	8.2	7.8
Salad Dressing	8.4	2.9	9.3	3.6	3.8	1.9	4.1	3.9	4.8
White Bread	5.1	3.2	9.6	4.7	4.0	4.8	3.6	3.0	4.7
Fars, margarine		11.8	2.6	5.5	2.6	1.3	1.6	6.4	4.0
Processed meat	2.8	2.5	4.4	2.2	3.2	1.2	1.7	2.2	2.5
Eggs, Regular	2.6	1.4	2.6	3.4	2.1	1.3	2.5	1.7	2.2
Potato Chips	1.3	1.4	0.0	3.1	0.8	0.7	0.8	0.0	1.0
Crackers,	2.5	0.0	0.0	0.8	1.4	0.8	1.4	1.1	1.0
Vegetable shortening	0.0	0.0	0.0	3.3	1.1	0.0	0.9	2.6	1.0

**Table 10:** Percentual contribution of food groups to trans fats consumption (%).

Food Group	Argentina	Brazil	Chile	Colombia	Costa Rica	Ecuador	Peru	Venezuela	ELANS
Not processed meat	26.5	28.3	14.0	25.6	19.5	31.5	24.0	29.0	24.8
Breads	12.5	10.9	24.0	9.0	1.8	11.8	24.6	5.8	12.6
Cheese	7.1	4.6	6.4	3.2	6.6	11.8	5.3	19.0	8.0
Vegetable oils	5.1	3.1	6.0	9.1	8.2	7.6	10.1	6.3	6.9
Margarine	2.1	20.6	0.0	9.0	0.0	6.1	0.0	17.5	6.9
Butter	7.0	5.0	10.5	4.8	1.7	6.7	1.8	0.0	4.7
Cakes and cookies	9.2	0.0	4.9	0.0	11.8	1.6	3.5	2.8	4.2
Milk and milk products	0.0	4.9	2.4	7.4	2.6	3.2	4.6	2.5	3.4
Processed meat	2.7	1.4	4.3	3.4	9.2	3.4	0.0	1.4	3.2
Vegetable shortening	0.0	0.0	2.6	7.9	2.3	0.0	4.2	5.8	2.8

## Data Availability

The data presented in this study are available upon request from the corresponding author. The data are not publicly available due to privacy or ethical restrictions.

## Supplementary Materials

The following supporting information can be downloaded at: https://www.mdpi.com/article/10.3390/nu16223940/s1, Table S1: Mean of the w6/w3 ratio by country.
