# Supplementary material for: Contributions of Fat and Fatty Acids Intake to the Latin American Diet: Results of ELANS Study
Source: Nutrients. 2024 Nov 19;16(22):3940. doi: 10.3390/nu16223940 (PMC11597284; doi:10.3390/nu16223940)
Supplement: Supplementary file 1 [file nutrients-16-03940-s001.zip › nutrients-3307543-supplementary.docx]

Contributions of fat and fatty acids intake to the Latin American diet: Results of ELANS study

Lilia Yadira Cortés Sanabria, Marianella Herrera-Cuenca, Martha Cecilia Yépez García, Pablo Hernandez, Guillermo Ramírez, Maura Vásquez, Yaritza Sifontes, María Reyna Liria-Domínguez, Attilio Rigotti, Mauro Fisberg, Agatha Nogueira Previdelli, Irina Kovalskys, Maritza Landaeta-Jiménez, and Georgina Gómez

**Supplementary material**

To assess the imbalance in the intake of ω3 and ω6 fatty acids, the ω6/ ω3 ratio was calculated (Table S1). According to experts, the ideal ratio should be 4:1. In all the countries studied, ratios were higher than the recommendation, with statistically clearly marked differences between them (p-value < 0.001), which leads to the establishment of the following order:

- Venezuela with the lowest value in the region whose ratio, with 95% confidence, varies between 7.02 and 7.14.
- Peru, Colombia and Brazil had values relatively higher levels than Venezuela, where the ratio ranges between 8.03 and 8.12, with 95% confidence.
- Costa Rica (9.67 to 10.0), Chile (11.0 to 11.51), Ecuador (12.32 to 13.01) and Argentina (17.72 to 18.53) had the highest values, with significant differences between them.

Table S1. Mean of the w6/w3 ratio by country.

| **Country** | **n** | **Mean** | **Standard Error** | **95% CI** | |
| --- | --- | --- | --- | --- | --- |
|  |  |  |  | **Lower** | **Upper** |
| ELANS | 9218 | 10,19 | 0,05 | 10,09 | 10,29 |
| Argentina | 1266 | 18,12 | 0,21 | 17,72 | 18,53 |
| Ecuador | 800 | 12,66 | 0,18 | 12,32 | 13,01 |
| Chile | 879 | 11,26 | 0,13 | 11,00 | 11,51 |
| Costa Rica | 798 | 9,84 | 0,09 | 9,67 | 10,00 |
| Brazil, Peru, Colombia | 4343 | 8,08 | 0,02 | 8,03 | 8,12 |
| Venezuela | 1132 | 7,08 | 0,03 | 7,02 | 7,14 |
